# The role of agriculture in Nepal's economic development: Challenges, opportunities, and pathways for modernization

**DOI:** 10.1016/j.heliyon.2025.e41860

**Published:** 2025-01-09

**Authors:** Lokendra Nath Yogi, Tara Thalal, Sarada Bhandari

**Affiliations:** Institute of Agriculture and Animal Science, Tribhuvan University, Nepal

**Keywords:** Agriculture, Development, Employment, Productivity, Resilience

## Abstract

Nepal's economy is primarily dependent on agriculture, which generates a significant amount of GDP and jobs, particularly in rural areas. Despite its importance, the business still faces challenges from low productivity, traditional methods, inadequate access to advanced technologies, and increasing climate change sensitivity. These problems prevent agriculture from realizing its full potential to advance economic development, reduce poverty, and provide food security. Modernization requires increasing productivity through mechanization, expanding infrastructure, and improving market accessibility. Finding a balance between these advancements and the need to protect rural jobs is therefore very important. Developing agro-industries, integrating value-added product, and empowering women and youth in agriculture are all essential approaches for inclusive growth. Since agriculture would free up labor and resources for other sectors, it is possible that agriculture also pushed Nepal's industrialization. The significance of strategic investments and reforms in the agriculture sector for Nepal's sustained economic growth and poverty eradication is emphasized in this article.

## 1 Introduction

Over many years, agriculture has been an essential economic sector, especially in agricultural nations where it is vital to maintaining livelihoods, guaranteeing food security, and driving economic growth. Across the board, agriculture has been acknowledged as an important driver behind economic growth, particularly in the early phases of industrialization. It offers job opportunities, raw materials for companies, and foreign exchange revenues from exports in addition to contributing to the GDP. The Food and Agriculture Organization (FAO) estimates that agriculture contributes roughly 4 % of the world's GDP, however in underdeveloped nations it can contribute up to 25 % [1]. Nepal is one of several emerging nations where agriculture still serves as the main economic pillar. The major economic sector in Nepal is agriculture, which employs 60 % of the labor force and adds roughly 23.9 % to the country's GDP [2]. Notwithstanding its importance, Nepal's agricultural output is still comparatively low due to a number of problems such as dependence on antiquated techniques for cultivation, restricted access to contemporary technologies, and susceptibility to climate change [3]. The sector's ability to make greater contributions to economic growth has been limited by these problems. In the past, the agricultural industry has been essential to lowering poverty and promoting rural development. World Bank research is among the many studies that indicate GDP growth from agriculture is twice as effective in eliminating poverty as growth from other sectors [4]. This is so because the bulk of the impoverished in emerging nations are found in rural areas, where agriculture has a direct impact on their way of life. Consequently, it has been believed that investments in market access, infrastructure, and agricultural productivity are crucial for inclusive economic development.

**Fig. 1:**
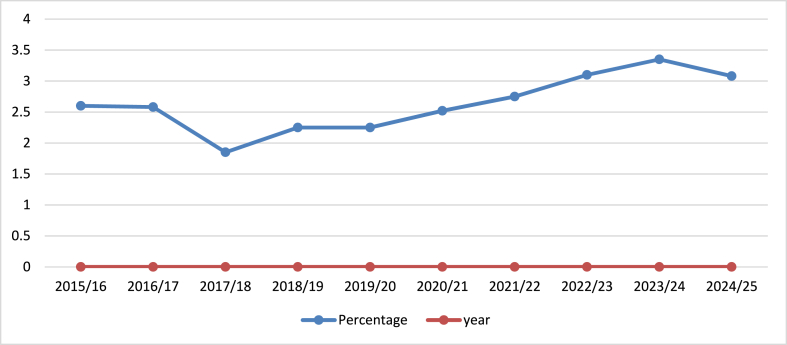
Budget distribution in agriculture sector (in percentage).

Moreover, agriculture acts as a foundation for the structural transformation of economies. In classical economic theories, such as the Lewis Model, agricultural surplus is considered critical for supporting industrialization by providing food, raw materials, and surplus labor [5]. This transition has been observed in many countries where industrial growth was preceded by agricultural modernization. However, in Nepal, the transition from agriculture to industrialization has been slow due to infrastructural deficiencies, fragmented landholdings, and poor access to markets and finance [6]. Nepal remains predominantly an agrarian society, with approximately 62 % of its population involved in agriculture, which accounts for about one-third of the national GDP; however, this contribution has seen a decline over the years, dropping from 36.64 % in the past to 23 % by 2022. Additionally, employment in agriculture has fallen from 73 % in 2005 to 62 % in 2021. Despite the agriculture sector's critical contribution to GDP and employment, budget allocation to the sector has remained relatively low over the years. As illustrated in Fig. 1, the share of the total national budget allocated to agriculture has fluctuated significantly. In 2015/16, agriculture received 2.6 % of the total budget, with the allocation holding steady in the subsequent year. However, from 2017/18, the percentage decreased to approximately 1.8 %, followed by a gradual increase in the following years. By 2023/24, the allocation had risen to 3.36 %, before experiencing a slight decline to 3.05 % in 2024/25. This trend underscores the inconsistent prioritization of agriculture despite its substantial role in the country's economy [7,8].

The primary objective of this article is to analyze the role of agriculture in Nepal's economic development, with particular emphasis on its current contributions, challenges, and potential for future growth. This article focuses on the economic impact of agriculture in Nepal, with a particular emphasis on its contributions to national GDP, employment, and poverty reduction. The scope includes an analysis of historical trends in agricultural development, the current challenges facing the sector, and the opportunities for growth through modernization and policy reform. The geographical focus is limited to Nepal, although references to global and regional trends in agriculture will be made for comparative purposes. The article draws on data from government reports, academic literature, and international organizations such as the World Bank and FAO to provide a comprehensive overview of the topic. The aim is to offer actionable insights that can inform policymakers, stakeholders, and researchers interested in enhancing the role of agriculture in economic development.

## 2 Methodology

This study employs a multi-method approach to analyze the contribution of agriculture to Nepal's economic growth, using secondary data from a variety of reputable sources. Government reports from the Central Bureau of Statistics (CBS) and the Ministry of Agriculture and Livestock Development (MoALD) of Nepal were used to assess agriculture's contribution to GDP and employment. In addition, data from international organizations such as the World Bank, Food and Agriculture Organization (FAO), and Asian Development Bank (ADB) were incorporated to provide both global and regional perspectives on Nepal's agricultural performance. To complement these secondary data sources, peer-reviewed research and academic publications were reviewed to identify key challenges in Nepal's agriculture sector, including low productivity, market access issues, and the impacts of climate change. While this study relies on secondary data due to resource constraints, its limitations are acknowledged. For instance, potential biases in reporting and the lack of granular, localized insights could influence findings. To address this, data were triangulated from multiple sources to enhance reliability. Future research could integrate primary data, such as household-level surveys, stakeholder interviews, and field-level case studies, to offer a more nuanced understanding of challenges such as credit access, market connectivity, and gender-based disparities.

The study also integrated qualitative data, such as policy evaluations and case studies, to gain a deeper understanding of the experiences of farmers and the effectiveness of agricultural policies. Additionally, quantitative data (e.g., GDP contribution, employment statistics, and agricultural production figures) were analyzed using descriptive methods to uncover trends and patterns over time. A comparative analysis was conducted to evaluate Nepal's agricultural development relative to other emerging agrarian economies, highlighting key factors such as access to credit, rural infrastructure, and technological adoption. These insights, combined with policy evaluations, provided a comprehensive view of Nepal's agriculture sector and its potential for future growth. All secondary data sources were properly cited and acknowledged, and ethical considerations, including the transparency of secondary data usage, were carefully observed. This approach provides a strong foundation for understanding macro-level trends and informs actionable policy recommendations while underscoring the need for future research based on localized primary data.

## 3 Agriculture as a contributor to Gross Domestic Product (GDP) in Nepal

**Fig. 2:**
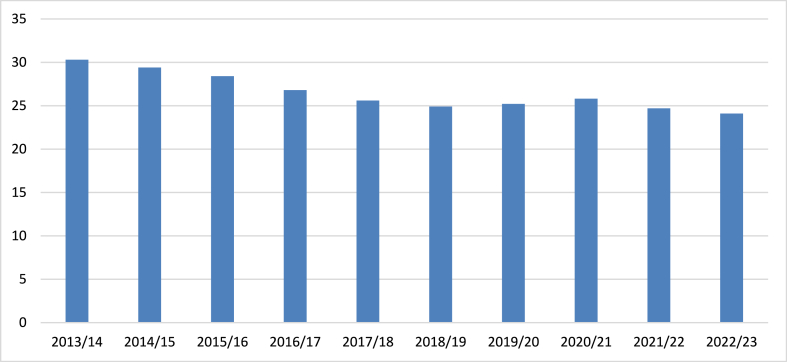
Contribution of agriculture sector to Gross Domestic product (GDP) in percentage.

In Nepal, agriculture is a vital sector, contributing approximately 27–30 % to the GDP and playing a crucial role in sustaining livelihoods, particularly as the global average is around 4 % and often exceeds 25 % in developing nations; while agriculture dominates, the manufacturing sector contributes about 10–15 % and is expected to grow as the economy develops, alongside a burgeoning services sector that accounts for 50–55 % of GDP. Despite its importance, agriculture faces significant challenges such as low productivity due to limited access to modern techniques, barriers to market access, and vulnerability to climate change, which jeopardizes food security and rural livelihoods. However, opportunities exist for modernization through technology, crop diversification to mitigate risks, the development of agro-processing industries for added value and income, and potential export markets for high-value products like organic fruits and vegetables, which could enhance the sector's contributions to the economy [9,10]. Fig. 2 presents the contribution of Nepal's agriculture sector to the national Gross Domestic Product (GDP) over a series of fiscal years. In 2013/14, the agriculture sector accounted for over 30 % of total GDP, marking its highest share during the period under review. However, this share experienced a gradual decline, reaching approximately 25 % by 2018/19, where it remained stable for the following year. In 2020/21, the contribution rose slightly to around 26 %, before decreasing again in 2021/22, falling below 25 %. By 2022/23, the sector's share had further reduced to approximately 24 %. This trend reflects a shifting economic structure, with other sectors progressively contributing more to the overall GDP [11].

## 4 Agriculture and employment in Nepal

**Fig. 3:**
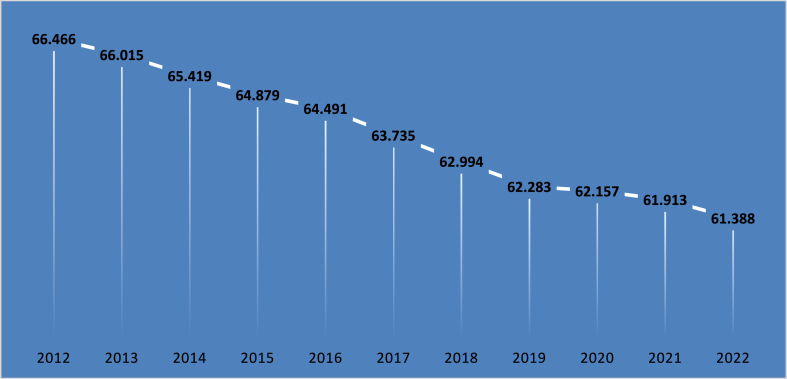
Employment in agriculture sector (percentage of total employment).

In Nepal, agriculture serves as a cornerstone of employment, engaging over 60 % of the workforce, particularly in rural areas where it plays a critical role in sustaining livelihoods and supporting overall food security; this sector not only provides direct employment through farming but also fosters indirect job creation in related industries such as food processing, packaging, transportation, and trade, which are vital for bolstering the rural economy [12]. However, the balance between providing jobs and enhancing productivity is a significant challenge, as many agricultural practices are characterized by low productivity due to factors such as subsistence farming, small landholdings, and limited access to modern tools. While there have been efforts to introduce mechanization and modern agricultural techniques, such as improved seeds and irrigation, these advancements may reduce the need for manual labor, potentially threatening rural employment levels. Thus, Nepal faces the critical task of improving agricultural productivity while ensuring that job opportunities are maintained or even created, as mechanization can free labor for other productive activities if coupled with skill development and alternative employment avenues. Additionally, given the prevalence of small-scale farming, it is essential to implement appropriate technologies that enhance productivity without completely displacing labor, as options like agro-processing and organic farming could provide labor-intensive alternatives that align with both Nepal's environmental needs and market demands [13,14]. Fig. 3 illustrates the percentage of total employment engaged in the agriculture sector over time. In 2012, approximately 66.47 % of the total workforce was employed in agriculture. However, this share has steadily declined over the years, with the proportion of people employed in agriculture decreasing each year. By 2022, the percentage had dropped to 61.39 %, reflecting a shift in employment patterns as other sectors have seen greater workforce engagement [15].

## 5 Gender and youth employment in agriculture

**Fig. 4:**
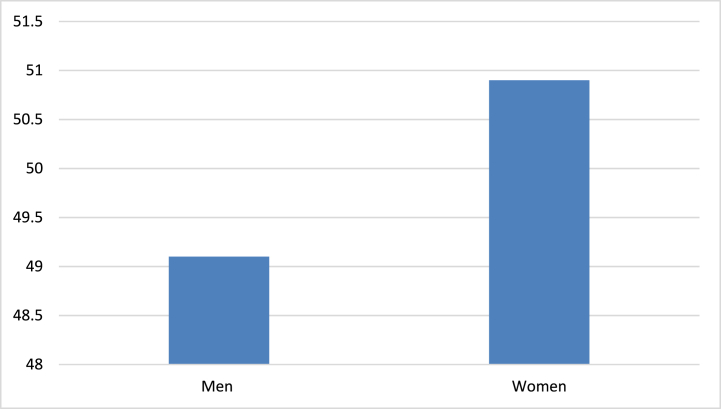
Illustrates the percentage of the total population engaged in agricultural work, categorized by gender.

Agriculture in Nepal is a vital source of employment for both women and youth, playing an essential role in the rural economy; women make up a significant portion of the agricultural workforce and are often seen as the backbone of Nepalese agriculture, yet they encounter various challenges, including limited access to land, resources, and decision-making power, which relegates many to low-paying, informal agricultural work with few opportunities for advancement. Empowering women through improved access to training, finance, and technology can enhance productivity and economic security for households, making it critical to address these disparities. On the other hand, while agriculture holds considerable potential for youth employment, many young individuals are migrating abroad or to urban centers in search of better opportunities, largely due to the perceived lack of prospects in traditional farming. To retain youth in agriculture, it is necessary to foster innovation, entrepreneurship, and the adoption of modern agribusiness models that appeal to the younger generation. Initiatives such as agri-entrepreneurship, organic farming, and agri-tourism can attract youth by incorporating technology—like digital platforms for market access—and sustainable farming practices that modernize the sector. Furthermore, government and non-governmental initiatives, including the Prime Minister's Agriculture Modernization Project and various youth-focused programs, have the potential to significantly enhance the participation of women and youth in agriculture; by expanding access to essential resources, skills training, and financial support, these efforts can increase engagement in productive agricultural activities, ultimately contributing to the growth and sustainability of Nepal's agricultural sector [16]. Agriculture remains the primary source of livelihood for a significant portion of Nepal's population, with 19.44 million Nepalis, out of a total population of 29.16 million, relying on farming. Among these individuals, 9.54 million (representing 49.1 %) are male, and 9.90 million (50.9 %) are female. Fig. 4 illustrates the gender distribution of the total population engaged in agricultural work, highlighting that women represent a slightly higher share (50.9 %) compared to men (49.1 %). This greater involvement of women in agriculture is largely attributed to factors such as male migration for work, traditional gender roles, limited employment opportunities for women, the prevalence of subsistence farming, and the socio-economic significance of agriculture in Nepal. This gendered reliance underscores the pressing need for policies that equip female farmers with improved resources, training, and technology to enhance productivity, food security, and rural economic growth. However, this reliance also exposes several challenges, as many women lack access to land ownership, financial support, and decision-making power, all of which hinder agricultural resilience and sustainable rural development [17].

## 6 Agriculture and food security in Nepal

Agriculture plays a critical role in ensuring food security in Nepal, where the majority of the population depends on subsistence farming for their livelihoods, yet challenges such as limited arable land, climate change, and outdated agricultural practices hinder food production and availability; agricultural development directly influences food security by ensuring the availability of staples like rice, maize, and wheat, with about 70 % of the population relying on agriculture for food and income, although food insecurity persists in certain regions due to low productivity and infrastructural challenges; government policies aimed at enhancing agricultural productivity, such as the Agriculture Development Strategy and the Prime Minister's Agriculture Modernization Project, have the potential to improve food security but face inefficiencies, especially in remote areas, compounded by uneven resource distribution and increasing dependency on food imports, which further threatens stability; thus, adopting sustainable farming practices, implementing climate-resilient agriculture, improving water management, and fostering community-driven initiatives are essential strategies to maintain long-term food security in the face of these challenges [18].

## 7 Agriculture and poverty alleviation in Nepal

Agriculture plays a vital role in poverty alleviation in Nepal, where a large portion of the population depends on farming for their livelihoods. By improving agricultural productivity, rural households can generate higher incomes, enhance food security, and reduce the risk of hunger and malnutrition, all of which are closely tied to poverty. Moreover, agricultural growth stimulates related sectors like food processing, transportation, and input supply, creating additional job opportunities and supporting broader rural economic development [19,20]. With strategic investments in infrastructure, modern farming techniques, and value chain integration, agriculture can become a powerful tool for reducing poverty in Nepal [21]. However, agriculture faces significant challenges that limit its potential impact on rural livelihoods. Many rural families rely on agriculture as their primary source of income, but low productivity, limited access to markets, and inadequate infrastructure constrain their earnings and overall well-being. Additionally, traditional farming practices and a lack of modern technology reduce efficiency, keeping many farmers trapped in poverty. While government and non-government initiatives have made some progress by promoting improved farming methods and offering support to small farmers, gaps remain.

Value chain development in key crops, such as rice and maize, can significantly contribute to poverty alleviation through targeted investments. For instance, integrating smallholder farmers into regional and national markets, supported by rural credit systems and improved post-harvest technologies, has proven successful in reducing poverty in countries like Vietnam and India. In Nepal, enhancing infrastructure such as irrigation systems, rural transportation networks, and cold storage facilities could yield similar benefits, improving income, employment opportunities, and food security for farmers and rural communities. A study by Teker et al. (2023) found that financial inclusion significantly influences economic growth, with a bidirectional relationship observed between financial inclusion and GDP per capita in both Turkey and Greece. The study also revealed a reciprocal relationship between financial inclusion and the Poverty Headcount ratio in both countries [22]. Addressing these issues through policies that expand market access, improve rural infrastructure, and provide financial resources could transform agriculture into a more effective tool for reducing poverty across Nepal.

## 8 Agriculture's role in industrialization in Nepal

Agriculture has played and continues to play a crucial role in the industrialization of Nepal. While agriculture dominates the country's economy, employing a large portion of the population, its development can provide the foundation for industrial growth by contributing labor, capital, and raw materials. Here's an analysis of agriculture's role in fostering industrialization in Nepal.

### 8.1 Structural transformation

Structural transformation refers to the transition from an agrarian-based economy to one that is industrial and service-oriented, with agriculture in Nepal playing a foundational role by providing labor and capital necessary for this shift; as modernization and technological advancements in agriculture lead to increased productivity and reduced reliance on manual labor, the resulting surplus workforce can migrate to the industrial sector, where emerging job opportunities in manufacturing, construction, and services are emerging, echoing historical patterns seen in countries like the UK and Japan [23]. Furthermore, while agricultural surpluses generated through enhanced productivity not only contribute to capital formation for industrial investment but also create essential backward and forward linkages between agriculture and industry—where industries supplying fertilizers, machinery, and irrigation support agricultural productivity, and agro-processing industries enhance the value of agricultural products, thus fostering economic growth, creating employment, and driving overall industrial development in Nepal [24].

### 8.2 Technological innovation in agriculture

Innovation in agriculture is crucial for enhancing productivity, stimulating economic growth, and supporting industrialization, as technological advancements have the potential to significantly increase agricultural output, lower costs, and create new industries linked to farming. For instance, precision farming, which utilizes technology such as sensors, GPS, and data analytics, can optimize resource use like water and fertilizers, thereby improving efficiency and productivity, while its adoption in Nepal could lead to substantial benefits by allowing farmers to produce more with fewer inputs, thus freeing up labor for industrial activities [25,26]. Enhancing irrigation infrastructure and mechanizing farming processes can dramatically boost productivity by stabilizing production and enabling higher incomes for farmers, which can facilitate the movement of surplus labor into industrial sectors. The rise of digital agriculture offers opportunities for connecting farmers to markets and providing real-time information, thereby increasing efficiency in the supply chain and strengthening agricultural links to industries like agro-processing [[27], [28], [29]]. Fostering innovations centered on sustainability, such as organic farming and agroforestry, not only enhances long-term productivity and environmental conservation but also aligns with Nepal's growing eco-tourism and organic export markets, creating pathways for related industries to flourish.

### 8.3 Value addition and agro-based manufacturing for Nepal's industrial growth

In Nepal, enhancing agricultural productivity through value-added processes and integrating agriculture with industrial manufacturing can substantially drive economic growth. Value addition in agriculture which includes processing fruits, vegetables, dairy, and herbal products holds tremendous potential. For instance, developing agro-industries focused on processing local crops like apples, ginger, cardamom, and medicinal herbs can elevate Nepal from being a supplier of raw materials to a competitive producer of finished goods. This approach can increase farmers' incomes, create employment opportunities in rural areas, and contribute to export earnings. Agro-based manufacturing industries, such as food processing, organic product packaging, and agro-pharmaceuticals, are key to transforming Nepal's agriculture sector. By establishing backward and forward linkages for example, connecting farmers with suppliers of fertilizers and equipment while ensuring processed goods reach both local and export markets these industries can strengthen the economy. Additionally, developing small-scale processing units in rural areas can decentralize industrial growth, providing job opportunities directly within farming communities and reducing dependency on imports for essential agricultural products. Combining value addition with agro-based manufacturing thus aligns with Nepal's economic goals by fostering a resilient agricultural sector that not only supports industrial growth but also advances rural development [30].

## 9 Challenges and barriers to agricultural development in Nepal

The primary sector of Nepal's economy is agriculture, but it faces several obstacles, such as the consequences of climate change, limited access to capital and markets, and ineffective policies that jeopardize the sector's sustainability and growth. Due to their high debt levels, unpredictable weather patterns, and restricted access to contemporary technology, smallholder farmers are especially vulnerable and experience productivity losses. Their prospects to expand their business are further limited by inadequate infrastructure and deficiencies in market knowledge. Furthermore, these problems are made worse by incompetent institutions and ineffectual policies, including problems with land tenure and ineffective subsidy schemes deterring investment. The long-term viability of Nepal's agricultural industry depends on addressing these obstacles through climate-smart practices, improved finance access, and improved governance.

## 10 Future outlook for agriculture in economic development in Nepal

Driven by technological breakthroughs like digital agriculture, mechanization, and the field of biotechnology Nepal's agriculture sector has enormous potential for expansion and modernization. These technologies can solve urgent issues like labor shortages, wasteful resource usage, and limited market access while greatly increasing productivity, advancing sustainability, and enhancing climate resilience. Climate change may improve land suitability for wheat cultivation in Nepal's middle and high-altitude regions, supporting agricultural growth, though risks from heat waves and droughts still pose challenges [31]. Agriculture can play a pivotal role in driving Nepal's economic development by attracting investment, improving access to financial resources, and fostering sustainable growth through modern farming practices [32]. Nepal can fortify its agricultural base and ultimately promote more equitable and sustainable economic growth by adopting these technologies.

## 11 Global and regional trends

Understanding global and regional agricultural trends is essential for shaping Nepal's agricultural strategy and ensuring competitiveness, as the global focus shifts toward sustainable, climate-resilient practices like organic farming and agroforestry, which can help Nepal tap into niche markets, while regional integration with India and China presents opportunities for expanding exports of high-value crops such as tea, spices, and medicinal herbs; at the same time, urbanization-driven changes in food consumption patterns highlight the potential for Nepal's agro-processing industry to grow and meet the demand for value-added products, provided that trade policies, infrastructure, and value chain improvements are in place to capitalize on these opportunities and simultaneously address food security concerns [33]. By embracing global and regional trends in financial inclusion, Nepal's agricultural sector has the potential to unlock substantial economic growth, fostering a resilient and sustainable economy that aligns with broader development goals.

## 12 Policy recommendations

To maximize agriculture's contribution to Nepal's economic development, policy reforms and strategic initiatives should focus on investing in agricultural R&D to develop climate-resilient crop varieties and technologies, improving rural infrastructure and market access to reduce post-harvest losses, facilitating targeted credit schemes and financial literacy programs for smallholder farmers, strengthening agricultural extension services to provide farmers with modern farming techniques and climate adaptation strategies, promoting climate-smart agriculture through incentives for sustainable practices, supporting agro-processing industries and integrating farmers into value chains to boost incomes and job creation, and encouraging public-private partnerships to attract investments, modernize the sector, and enhance market linkages. Nepal can look to successful examples like the Netherlands, where advanced agriculture technology and efficient supply chains have made it a leading global exporter, or Isreal, which transformed its arid land into productive farmland through irrigation innovation. China improved food security and rural incomes by providing subsidies for high yield seeds and fertilizers, built extensive irrigation system, and connecting farmers to national and international markets. To further strengthen agricultural development in Nepal, expanding the reach of cooperative banks and credit unions, similar to successful models in countries like Poland, could play a crucial role in promoting financial inclusion, supporting local economic growth, and providing rural populations with greater access to financial services, ultimately enabling smallholder farmers to invest in modern agricultural technologies and improve their livelihoods [34]. To enhance financial inclusion and support agricultural and rural development in Nepal, policymakers should consider adopting models from successful international examples, such as microfinance and non-financial institutions (NFIs), which have proven effective in promoting economic growth and sustainability. By leveraging new technologies and increasing investment in FinTech, Nepal could expand the role of NFIs in rural areas, improving the social and financial performance of smallholder farmers and entrepreneurs, ultimately driving inclusive growth and poverty reduction [35]. To foster long-term economic stability and growth, it is crucial to carefully balance credit funding with the regional Gross Regional Product (GRP), as unchecked investment aimed at fulfilling immediate needs may undermine macroeconomic stability. This highlights the importance of distinguishing between the routine financial mediation of business processes and the more strategic financing of development projects, which drive sustained economic progress. Moreover, enhancing financial intermediation through improved accessibility to financial services, creating favorable legal and infrastructural conditions, and expanding the role of non-bank financial institutions (NFIs) can reduce financial repression in underserved regions, ultimately promoting inclusive economic growth and stability [36]. To promote more equitable economic outcomes, policymakers should consider the distinctive role of cooperative banks in mitigating income inequality, particularly in smaller municipalities, by safeguarding their unique operational models and ensuring their continued presence and viability, as these banks have proven to reduce income disparities in the aftermath of economic crises [37]. By adopting similar policies, Nepal can enhance agricultural productivity, improve rural livelihoods, and contribute significantly to economic growth.

## 13 Discussion

More than sixty percent of the workforce is employed in agriculture, which continues to be a vital component of the Nepalese economy, contributing significantly to GDP. Although its significance, the industry confronts significant obstacles such as low production, antiquated farming methods, restricted availability of contemporary equipment, and susceptibility to climate change. Even though many people make their living from agriculture, the industry's shrinking GDP share emphasizes the need for modernization through mechanization, improved infrastructure, and market access. Additionally, there is an urgent need to develop jobs in value-added products and agro-industries, as well as to strike a balance between employment and productivity improvements, especially in rural areas. Enhancing agricultural production can increase food security and reduce poverty, especially as subsistence farming remains popular. Agriculture can significantly drive Nepal's economic development by improving regional socio-economic stability, fostering sustainable agricultural practices, and enhancing local governance and transparency, which collectively contribute to effective policy-making and long-term growth [38]. In order to empower women and young people in agriculture, specific programs that offer technical access, financial support, and training are required. Furthermore, as productivity rises, agriculture has the ability to propel industrialization in Nepal by creating agro-processing firms and freeing up labor and resources. In conclusion, agriculture's full potential may be realized through modernization and wise investments, which will promote economic growth, combat poverty, and guarantee long-term food security.

## 14 Conclusion

Agriculture remains central to the livelihoods of most Nepalis and continues to play a significant role in contributing to GDP and employment. Yet, the sector faces substantial challenges, including low productivity, reliance on traditional practices, limited access to technology, and vulnerability to climate impacts. Unlocking agriculture's full potential to reduce poverty and fuel economic growth requires strategic investments and policy reforms that focus on modernization. Investments in key areas such as rural infrastructure, irrigation, mechanization, and digital tools for farm management will be essential for driving productivity and resilience. Targeted reforms to improve access to markets, financial services, and training for farmers, especially women and young people, are crucial to making these gains inclusive.

To ensure that growth in agro-industries and value-added sectors supports job creation rather than displacing rural employment, policies must also prioritize the development of rural enterprises and cooperatives. Empowering marginalized groups within the agricultural workforce can lead to sustainable sector growth and foster inclusive rural development. Furthermore, agriculture holds significant potential for advancing Nepal's industrialization by releasing labor and capital for other sectors through productivity gains. Through strategic investments, effective reforms, and collaborative public-private partnerships, Nepal can transform its agricultural sector into a driver of long-term food security, inclusive economic growth, and sustainable poverty reduction.

## CRediT authorship contribution statement

**Lokendra Nath Yogi:** Writing – review & editing, Writing – original draft, Supervision, Resources, Methodology, Investigation, Data curation. **Tara Thalal:** Conceptualization. **Sarada Bhandari:** Resources.

## Data availability

The data for this study was collected from government papers, including those from the Ministry of Agriculture and Livestock Development (MoALD) and the Central Bureau of Statistics (CBS), providing insights into agriculture's contributions to GDP and employment. In addition, data from global institutions like the World Bank and the Food and Agriculture Organization (FAO) were employed for the purpose of comparative analysis. Scholarly publications provided background information on opportunities and problems facing Nepal's agriculture industry.

## Declaration of competing interest

The authors declare that they have no known competing financial interests or personal relationships that could have appeared to influence the work reported in this paper.
